# Repetitive sequence analysis and karyotyping reveals centromere-associated DNA sequences in radish (*Raphanus sativus* L.)

**DOI:** 10.1186/s12870-015-0480-y

**Published:** 2015-04-18

**Authors:** Qunyan He, Zexi Cai, Tianhua Hu, Huijun Liu, Chonglai Bao, Weihai Mao, Weiwei Jin

**Affiliations:** Institute of Vegetables, Zhejiang Academy of Agricultural Sciences, Hangzhou, 310021 China; National Maize Improvement Center of China, Beijing Key Laboratory of Crop Genetic Improvement, China Agricultural University, Beijing, 100193 China

**Keywords:** Radish, Repetitive DNA, Satellite, Karyotyping, CENH3, Centromere

## Abstract

**Background:**

Radish (*Raphanus sativus* L., 2n = 2x = 18) is a major root vegetable crop especially in eastern Asia. Radish root contains various nutritions which play an important role in strengthening immunity. Repetitive elements are primary components of the genomic sequence and the most important factors in genome size variations in higher eukaryotes. To date, studies about repetitive elements of radish are still limited. To better understand genome structure of radish, we undertook a study to evaluate the proportion of repetitive elements and their distribution in radish.

**Results:**

We conducted genome-wide characterization of repetitive elements in radish with low coverage genome sequencing followed by similarity-based cluster analysis. Results showed that about 31% of the genome was composed of repetitive sequences. Satellite repeats were the most dominating elements of the genome. The distribution pattern of three satellite repeat sequences (CL1, CL25, and CL43) on radish chromosomes was characterized using fluorescence in situ hybridization (FISH). CL1 was predominantly located at the centromeric region of all chromosomes, CL25 located at the subtelomeric region, and CL43 was a telomeric satellite. FISH signals of two satellite repeats, CL1 and CL25, together with 5S rDNA and 45S rDNA, provide useful cytogenetic markers to identify each individual somatic metaphase chromosome. The centromere-specific histone H3 (CENH3) has been used as a marker to identify centromere DNA sequences. One putative *CENH3* (*RsCENH3*) was characterized and cloned from radish. Its deduced amino acid sequence shares high similarities to those of the CENH3s in *Brassica* species. An antibody against *B. rapa* CENH3, specifically stained radish centromeres. Immunostaining and chromatin immunoprecipitation (ChIP) tests with anti-BrCENH3 antibody demonstrated that both the centromere-specific retrotransposon (CR-Radish) and satellite repeat (CL1) are directly associated with RsCENH3 in radish.

**Conclusions:**

Proportions of repetitive elements in radish were estimated and satellite repeats were the most dominating elements. Fine karyotyping analysis was established which allow us to easily identify each individual somatic metaphase chromosome. Immunofluorescence- and ChIP-based assays demonstrated the functional significance of satellite and centromere-specific retrotransposon at centromeres. Our study provides a valuable basis for future genomic studies in radish.

**Electronic supplementary material:**

The online version of this article (doi:10.1186/s12870-015-0480-y) contains supplementary material, which is available to authorized users.

## Background

Repetitive DNAs, including transposable elements and tandem repeats, are the major components of the genomic sequence and the most important factors in genome size variations in higher eukaryotes [1-3]. Based on the mechanism of transposition, transposable elements can be divided into two classes, transposons and retrotransposons. The majority of these elements in plant genome are long terminal repeat (LTR) retrotransposons and most of them are dispersed throughout all chromosomes [4,5]. Tandem repeats consist of large number of repeat units and are usually found in centromeres, pericentromeres or telomeres [6]. Tandem repeats are good cytogenetic markers for chromosome identification and molecular karyotyping [7].

Centromeres are specialized regions on chromosomes where centromeric protein and spindle microtubules attach via the kinetochore and typically contain large arrays of satellite repeats and/or retrotransposon-related repetitive sequences in eukaryotes [8,9]. They are essential for proper chromosome segregation during mitosis and meiosis. Although the function of centromeres is conserved in organisms, centromeric repeats appear to evolve rapidly [10]. Satellite repeats go through rapid evolution and significant variation between closely related species or even among different chromosomes of the same species [11-14]. Centromeric regions are comprised of repetitive sequences in most species, suggesting that those sequences play important roles in centromere function [15]. Centromeres are universally marked by the presence of a centromere-specific histone H3 (CENH3, called CENP-A in human), that replaces canonical histone H3 in centromeric nucleosomes to form functional centromeres [16]. CENH3 is a good marker to identify the core centromeric sequences by chromatin immunoprecipitation (ChIP) with an anti-CENH3 antibody [11,17,18].

Radish (*Raphanus sativus* L., 2n = 2x = 18), belonging to the family *Cruciferae*, is an important vegetable crop especially in eastern Asia. Radish root contains various nutritions which play a part in strengthening immunity [19,20]. Radish is a healthy vegetable and is popular in many dishes. Although radish is a significant vegetable crop, it still lacks cytogenetic analysis. Location of 5S rDNA loci and 45S rDNA loci were confirmed via FISH mapping [21,22]. These two sequences are located at the pericentromeric heterochromatin regions. A few studies of the radish repetitive DNAs were previously reported. First an alphoid-like satellite repeat in radish was found in 1986 [23]. It was a big step to get the draft sequences of the Japanese radish ‘Aokubi’, with a long and thick root, for the study of repetitive elements. It has been estimated that the genome size of the radish is 530 Mb [24] and about 26.6% of the genome is made of various DNA repeats. The transposons and retrotransposons were characterized [25]. Nevertheless, up to now, understanding of the repetitive sequences of radish is still not sufficient, especially for the tandem repeats. In this study, 5Gb of sequence data was used to analyze the repetitive elements of radish. We found three types of tandem repeats (CL1, CL25, and CL43) in the radish genome. An integrated metaphase chromosome karyotype was established using tandem repeats (CL1 and CL25), along with rDNAs as probes. The coding sequence of *CENH3* of radish was identified. Immunostaining and chromatin immunoprecipitation tests demonstrated that both CR-Radish and CL1 are associated with RsCENH3 proteins in radish.

## Results

### Composition of the repetitive sequences in the radish genome

5Gb sequencing data, which amounts to 4.8× coverage of the radish genome, was obtained from the HiSeq2000 platform. RepeatExplorer, a Graph-based clustering and characterization of repetitive sequence utilities was used for analyzing repetitive elements of the genome. 174 clusters were generated with cluster size threshold of 0.01%, and clusters which were annotated putative mitochondrial and plastid contaminations were removed. Finally, 144 clusters were used for calculating genome proportions (see Additional file 1). The genome proportions of each type of repetitive DNA are shown in Table 1. About 30.73% of the genome is repetitive DNAs. According to our results, it has different repetitive DNA types: retrotransposons (including Copia, Gypsy, and LINE/SINE), transposons (including hAT, Mutator, DNA/CMC-EnSpm, and Tc1-Mariner), rDNA and satellites. Satellite repeats, which occupy 12.93% of the genome, make up the most dominant part of the repetitive DNAs in radish. The majority of retrotransposons are Ty1/Copia and Ty3/Gypsy retrotransposons, with genome proportions of 5.81% and 4.88%, respectively. The genome proportion of transposons is only 1.41% and the most abundant transposon is hAT, with a 0.93% genome proportion. Estimation of rDNA elements abundance showed that they comprise 4.32% of the genome.

**Table 1:** **Repeat elements and their proportions in radish**

**Elements**	**GP (genome proportion, %)**
**Retrotransposon**	**11.05**
**Copia**	**5.81**
**Gypsy**	**4.88**
**LINE/SINE**	**0.33**
**Unclassified LTR**	**0.03**
**Transposon**	**1.41**
**hAT**	**0.93**
**Mutator**	**0.27**
**DNA/CMC-EnSpm**	**0.10**
**Tc1-Mariner**	**0.11**
**rDNA**	**4.32**
**Satellite**	**12.93**
**Unclassified**	**1.02**
**Total**	**30.73**

### Identification of subtelomeric repeats and centromeric repeats in radish

**Figure 1:**
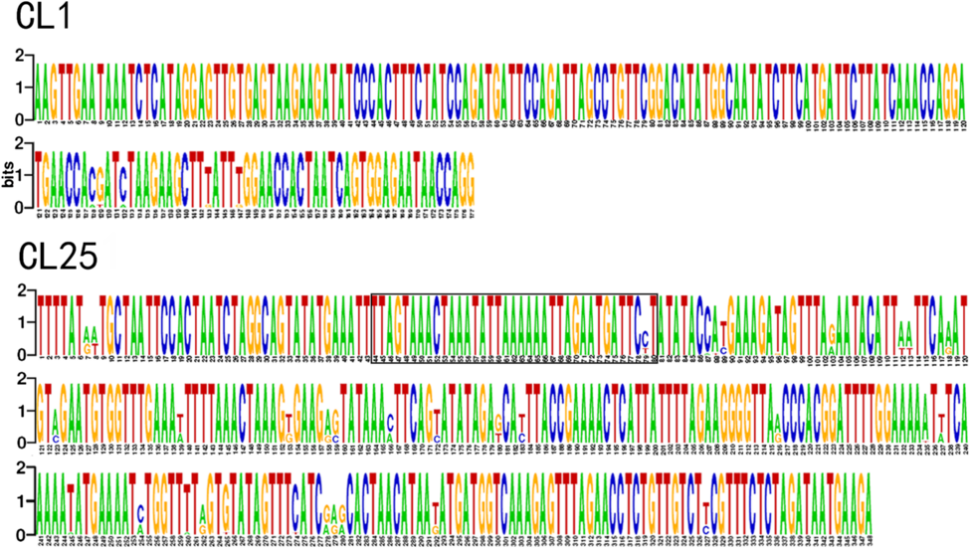
Consensus sequence of CL1 and CL25 repeats. The black rectangle outlines the positions in the sequence that share similarities to *C. elegans*.

In addition to 5S rDNA and 45S rDNA, three tandemly organized repeats (CL1, CL25 and CL43) were identified by bioinformatics analysis of the sequencing data. The CL1, CL25 and CL43 repeats were estimated to make up 12.32%, 0.44%, and 0.17% of the genome, respectively. CL43 is a telomeric repeat, consisting of a 7 bp monomer (TTTAGGG, the same as the *Arabidopsis* telomere sequence), located at both ends of chromosomes (see Additional file 2). PCR of CL1 and CL25 resulted in a ladder like pattern for tandemly organized repetitive units. To ascertain the size of the monomers of CL1 and CL25, specific primers were designed for amplifying these two repeats and then sequenced. According to sequencing results, CL1 consists of ~177 bp monomers (Figure 1), which is almost exactly the same as the size of the alphoid-like satellite repeat reported by Grellet [23]. Searching GenBank and PlantSat databases revealed high similarities to centromeric tandem repeats centBr1 and centBr2 from *Brassica* species (~80% identity over 177 bp) [26] and satellite sequences from *Sinapis alba* (~78% identity over 165 bp) [27]. The CL25 repeat is characterized by a ~348 bp monomer unit (Figure 1) and is a newly found satellite. Similar to CL1, the CL25 sequences shared high similarities to *Brassica* species (~78% identity over 348 bp). In addition, a small part (the black rectangle outlined region in Figure 1) of the CL25 sequence present in the *C. elegans*.

**Figure 2:**
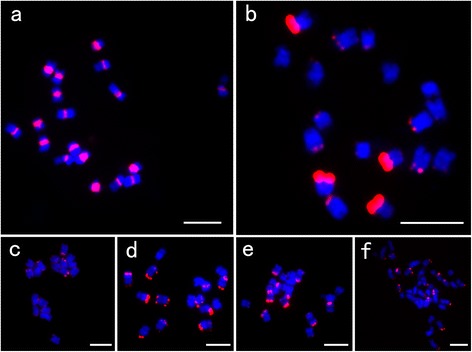
FISH mapping of CL1 and CL25 repeats. **(a)** FISH mapping of CL1 in radish; **(b-f)** FISH mapping of CL25; **(b)** radish; **(c)** *B. rapa*; **(d)** *B. nigra*; **(e)** *B. oleracea*; **(f)** *B. napus*. Bars = 5 μm.

FISH result showed that CL1 is located at the main primary constrictions and CL25 appears at the subtelomeric regions (Figure 2a-b). On account of CL25 sharing a high similarity to *Brassica* species, we speculated that CL25 should have a specific distribution pattern in these species. FISH mapping of CL25 repeats was performed on metaphase chromosomes of several *Brassica* species, including *B. rapa* (A genome), *B. nigra* (B genome), *B. oleracea* (C genome), and *B. napus* (AACC), which are close relatives to radish (Figure 2c-f). Overall, CL25 appeared at subtelomeric regions for all the detected species, although different species have various numbers and varied intensities of signals. Intensities of signals are relatively weak in *B. rapa*, and strong in *B. oleracea*. A different distribution pattern was detected in *B. nigra* with strong signals on 4 pairs of chromosomes and weak signals on 2 pairs of chromosomes. Therefore the CL25 repeat is an ancient repeat which appeared before the differentiation of tribe *Brassiceae* and radish. It has maintained its subtelomeric positions in all detected *Cruciferae* species.

### Karyotyping analyses of radish

**Figure 3:**
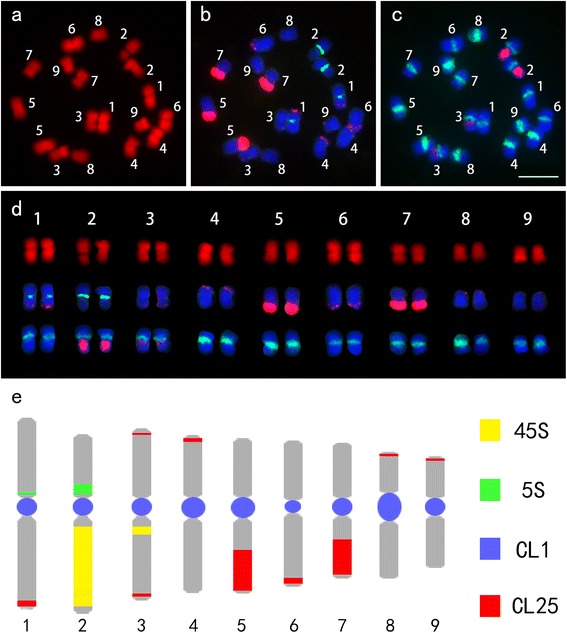
The karyotype and ideograph for radish mitotic metaphase chromosomes. **(a)** The mitotic metaphase chromosomes (numbered from 1 to 9) were counterstained with DAPI and pseudocolored in red; **(b)** FISH with the probe of CL25 (red) and 5S rDNA (green); **(c)**. The same spread was reprobed with the probe of CL1 (green) and 45S rDNA (red); **(d)** Individual chromosomes were separated from Figure (a-c) and listed according to their order; **(e)** Ideogram showing the position and intensity of CL25 (red) and 5S rDNA (green), CL1 (blue) and 45S rDNA (yellow). Bars = 5 μm.

Given the lack of DNA markers for FISH analysis, detailed molecular karyotype analyses of radish have not yet been conducted. Repeats identified in this study provide good markers for karyotyping analysis. Sequential FISH using repetitive DNA sequences (CL1, CL25, 5S rDNA and 45S rDNA) as probes were performed to identify radish chromosomes (Figure 3a-c). The CL1 signals appeared at the middle of all of the chromosomes with varied intensities. The CL25 signals were located at one arm of chromosomes 1, 4, 5, 6, 7, 8, and 9, and both arms of chromosome 3 with one pairs of signals. Signals were large and strong on chromosomes 5 and 7, but weak on chromosomes 1, 3, 4, 6, 8 and 9. Two pairs of 5S rDNA signals were detected, and one pair of strong signals were located at the peri-centromeric region of the short arm of chromosome 2, and the other pair of weak signals appeared at the peri-centromeric region of the short arm of chromosome 1. Interestingly, we detected 3 pairs of 45S rDNA signals in early generations, which is the same as Koo’s results [22]; however, only 2 pairs of the signals were detected 3 generations later (see Additional file 3). Seeds from the new generation were used for karyotyping analysis (2 pairs of signals). In our study, the signals of 45S rDNA were located at the long arms of chromosomes 2 and 3. Using satellite repeats (CL1 and CL25) combined with rDNAs as FISH probes, we distinctly identified individual somatic chromosome by the position and intensity of their signals (Figure 3d). An integrated ideogram of radish metaphase chromosomes is shown in Figure 3e.

### Cloning of *CENH3*

To identity *CENH3* in radish, we searched NCBI using the blastn program (Nucleotide collection, nr/nt) with the *BrCENH3* complementary cDNA sequence (GenBank accession number GU166737.1) as the query. Two radish CENP-A gene sequences (AB299183.1 and AB299184.1) were identified. These two putative *CENH3* open reading frames share high similarity with a small gap and some SNPs. Based on these two sequences, specific primers were designed to isolate the complete *RsCENH3* coding region from radish plants. According to cDNA sequencing results, three transcripts were detected: a 635 bp length of transcript (1/20), a 513 bp length of transcript (1/20), and the majority 537 bp length of transcript (18/20). To analyze the intron/exon structure of *RsCENH3*, the full length of genomic DNA sequence of *RsCENH3* was amplified using the same primers. On the basis of genomic DNA results, only one type of DNA sequence was found, which has a total length of 1415 bp. This sequence shares 100% identity to the AB299183.1 and is comprised of nine exons and eight introns. By comparison with the full length genomic DNA sequence, a 635 bp length of transcript transformed from the third intron into an exon, a 513 bp length of the transcript has a deletion from part of the forth exon, and the major transcript is 537 bp. Considering the translation, alignment to other plant *CENH3s,* and the proportion of these transcripts, we deemed that the small number of transcripts were produced by mis-splicing from the same loci and the *CENH3* comprises an open reading frame (ORF) of length 537 bp encoding a predicted 178-amino acid (Aa) protein.

**Figure 4:**
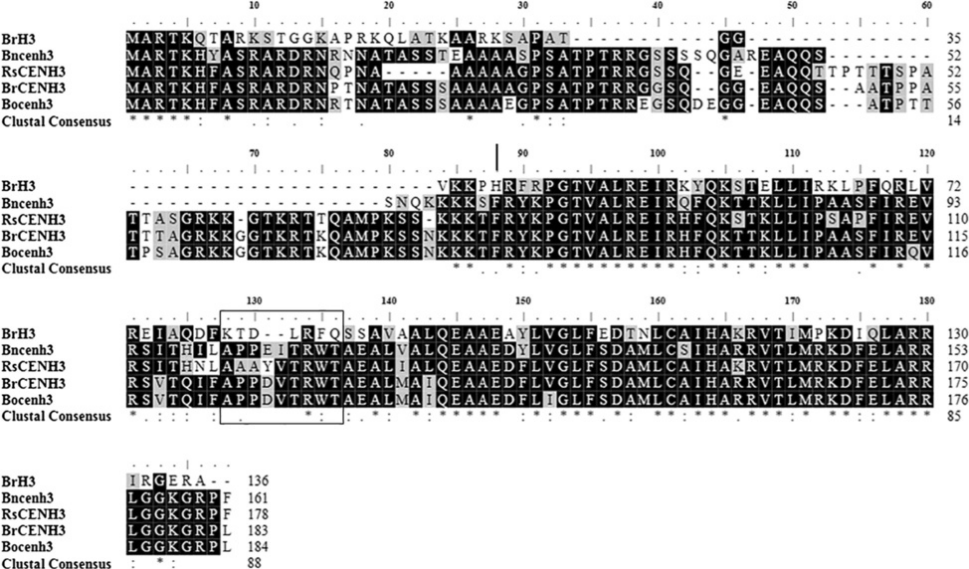
A multiple alignment of CENH3 sequences. A multiple alignment of radish (RsCENH3), *Brassica rapa* (BrCENH3), *Brassica oleracea* (BoCENH3), *Brassica nigra* (BnCENH3) homologs and *Brassica rapa* H3 (BrH3). A black rectangle indicates the position of loop1 region. The right side of the vertical bar is the N-tail region and left side is the histone fold domain.

Multiple sequence alignment revealed that RsCENH3 shares high identities with CENH3 from *Brassica* species, 77% identity with BrCENH3, 64% with BnCENH3, and 74% with BoCENH3. Several prominent features of the deduced RsCENH3 in comparison with those CENH3s and canonical histone H3 are as follows (Figure 4). A longer and more divergent N-terminal tail is present in the deduced *RsCENH3* sequence (178 amino acids in total) that is not alignable to BrH3 (136 amino acids in total). Each of the predicted proteins encoded a histone fold domain with similarities to histone H3. The loop 1 region in the histone fold domain is longer than that of canonical histone H3s (nine amino acids as opposed to seven for BrH3). All of these findings demonstrate that the sequence identified is an authentic CENH3 homolog in radish.

### DNA sequences associated with RsCENH3

**Figure 5:**
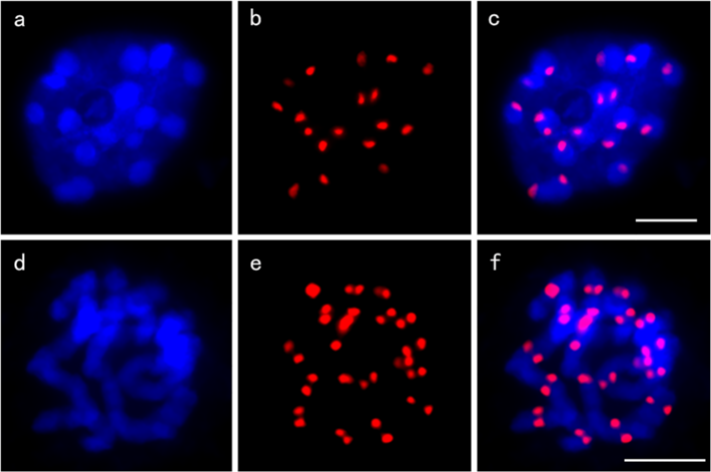
Anti-BrCENH3 antibody staining in mitotic cells of radish. RsCENH3 localization (red) on somatic interphase cell **(a**-**c)** and metaphase chromosomes **(d**-**f)**. Bars = 5 μm.

A *B. rapa* -derived CENH3 antibody (anti-BrCENH3) was previously used to confirm CENH3-associated centromeric sequences in different *Brassica* species [28]. Based on the similarities of CENH3’s sequence between radish and *Brassica* species, we speculated *B. rapa* -derived CENH3 antibody should recognize the RsCENH3 protein at core centromeres in radish. To confirm whether it recognizes the RsCENH3 protein, we applied an immunofluorescence assay on somatic cells of radish with the anti-BrCENH3 antibody. Signals appeared at the centromeric regions of all 18 metaphase chromosomes (Figure 5d-f). In interphase cells, RsCENH3 signals were located at the edge of the DAPI intensively stained heterochromatic regions (Figure 5a-c). It showed that the antibody also could recognize RsCENH3.

**Figure 6:**
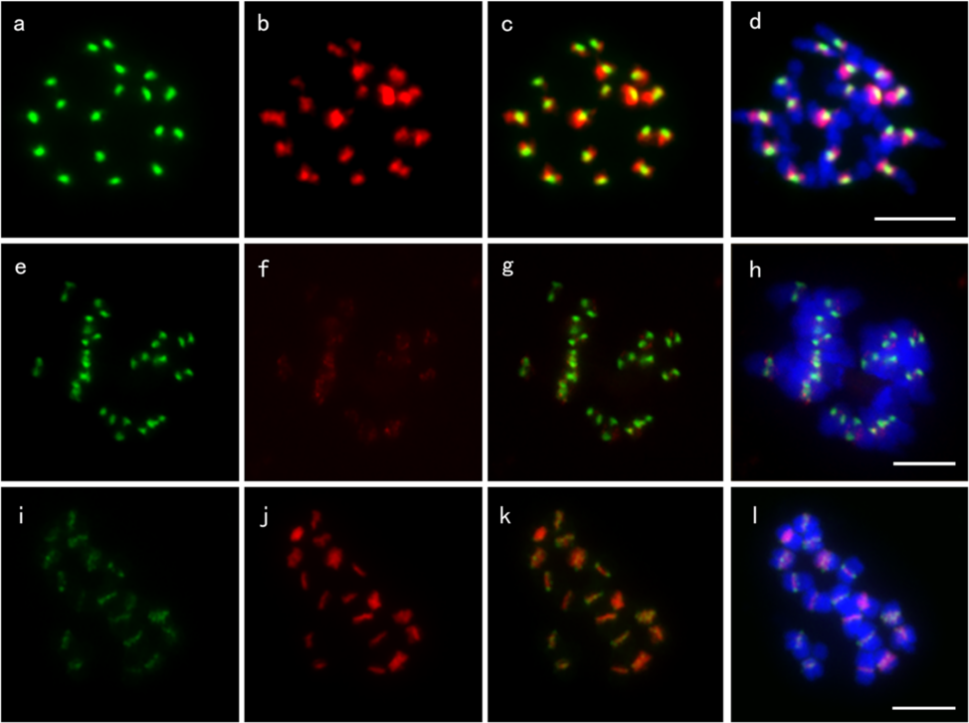
Sequential localization of the anti-BrCENH3 antibody and centromeric repeats on radish. **(a)** RsCENH3 localization at mitotic metaphase chromosomes of radish; **(b)** The same cell was hybridized with CL1; **(c)** Merged fluorescence signals from a and b; **(d)** Merged fluorescence signals from c and chromosomes; **(e)** RsCENH3 localization at mitotic metaphase chromosomes of radish; **(f)** The same cell was hybridized with CR-Radish; **(g)** Merged fluorescence signals from d and e; **(h)** Merged fluorescence signals from g and chromosomes; **(i)** CR-Radish localization at mitotic metaphase chromosomes of radish; **(j)** The same cell was probed with CL1; **(k)** Merged fluorescence signals from g and h; **(l)** Merged fluorescence signals from h and chromosomes. Bars = 5 μm.

It has been reported that CRB (Centromere-specific retrotransposons of *Brassica*) is a core centromeric sequences of *Brassica* species [28]. We also detected a CRB-like retrotransposon CL4, which represents 1.14% of the genome and was named CR-Radish in radish. To verify if the centromere-specific retrotransposon CR-Radish and the 177-bp satellite repeat CL1 were associated with RsCENH3 protein in radish, we performed an immunofluorescence assay followed by FISH on the same set of cells to detect the co-localization of BrCENH3 and centromeric DNA repeats. The size of RsCENH3 immuno-signals were relatively uniform among kinetochores while the size of CL1 signals were uneven among different chromosomes (Figure 6a-d). CL1 signals were overlapped with the RsCENH3 immuno-signals, although they were significantly larger than the RsCENH3 immuno-signals. These results suggest that only a limited part of the CL1 sequences are associated with the kinetochore complex. We also conducted anti-BrCENH3 immunostaining followed by FISH of the CR-Radish retrotransposon (Figure 6e-h). Different from that of CL1, CR-Radish signals were smeared and weak. As expected, the FISH signals overlapped with most of the immuno-signals. Therefore, we propose that the RsCENH3 protein is also associated with CR-Radish. Dual-color FISH showed most signals of CR-Radish and CL1 were co-localized, while the signals of CL1 were more concentrated than CR-Radish signals (Figure 6i-l).

**Figure 7:**
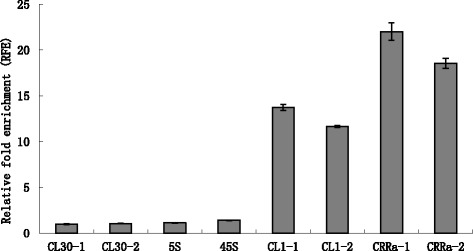
Sequences associated with RsCENH3. Relative fold enrichments of repeats obtained by ChIP with the anti-BrCENH3 antibody are shown for radish genomes. CL25 serves as a negative control; CR-Radish and CL1 were associated with CENH3.

To further confirm our immunostaining results, ChIP tests with the anti-BrCENH3 antibody were conducted to assess the association of CL1 and CR-Radish with RsCENH3. FISH using the ChIPed DNA as a probe showed high enhanced signals in the centromere regions of all radish chromosomes. In contrast, using mocked DNA as a probe showed no obvious signal (see Additional file 4) which indicates that the centromere sequences were specifically pulled down by the anti-BrCENH3 antibody in ChIP. The ChIP-qPCR was performed to verify the enrichment of putative centromeric repeats (Figure 7). Two specific primers, designed from different regions of CL1, CL25 and CR-Radish, were used to detect each fragment. The ChIP-qPCR was repeated three times using CL25 as extra-centromeric control. RFE value for CL25-1 was set at 1, and the RFE value of each sequence was normalized using the CL25-1 as a reference. The RFE of the non-centromeric control CL25-2, 5S rDNA, and 45S rDNA were low and similar to each other at 1.06 ± 0.04, 1.11 ± 0.03, and 1.41 ± 0.02, respectively (Figure 7). In contrast, the RFE of the CR-Radish fragments were as high as 22.02 ± 0.94 and 18.54 ± 0.53, respectively. Similarly, the RFE of the CL1 fragments were 13.71 ± 0.33 and 11.64 ± 0.11, respectively. These results indicate that CR-Radish and CL1 were significantly enriched in the ChIPed DNA. Therefore, CR-Radish and CL1 are associated with RaCENH3.

## Discussion

### Karyotype of radish

Up to now, studies on the radish genome were still limited and few cytogenetic and genomic studies were carried out [21,22,25]. Comparative analysis of rDNA and Rfk1 gene distribution in chromosomes of *Brassica* species and radish were carried through using FISH [21,22,29]. However, to our knowledge, a complete karyotype analysis that reliably distinguishes each chromosome of radish has not been reported. Chromosome identification is critical for cytological analyses, as well as subsequent studies in genomics, taxonomy, and the evolution of polyploidy, enabling an understanding of the relationship between visible landmarks and genetic or physical map features [30]. The somatic metaphase chromosomes of radish are small and lack feasible markers, which make adequate identification of radish chromosome pairs difficult. In this study, we used RepeatExplorer to conduct genome-wide analysis of repetitive sequences and obtained two useful cytogenetic markers (CL1 and CL25). Together with rDNAs, one or two signals were detected on each chromosome (Figure 3d). We are now able to easily identify all 9 somatic metaphase chromosomes by the position and intensity of FISH signals. In addition, an integrated metaphase chromosome karyotype was established (Figure 3e). Our study provides a valuable basis for future genomic studies.

### Dynamic nature of radish genome

Repetitive sequences contribute significantly to extraordinary genome size variation in higher plants [31,32]. Generally speaking, LTR-retrotransposons are the most abundant element of the genome, especially in big genome species, such as maize [5], wheat [33], and coix [34]. However, the majority of repetitive sequences are satellites, which make up 12.932% of the radish genome in our study. A similar high proportion of satellites were found in *C. rubella* and cucumber, in which more than 20% of the genome sequences are satellite repeats [35,36]. Ordinarily, several to dozens of types of satellite repeats are detected from a number of species [34,37-39]. In our study, only three satellite repeats were found in radish, including centromeric repeats, subtelomeric repeats and telomeric repeats (Figure 3). This is a typical pattern where the satellite DNA sequences are appear predominantly in the centromeric, pericentromeric and telomeric regions [40,41]. The dynamic evolutionary processes of satellite DNA may generate changes in its chromosomal location and distribution. Some satellite DNA families were found to be species-specific [42], while others were more conserved, and similar sequences may be isolated in closely related species [26,43]. In our study, we detected 3 pairs of 45S rDNA signals in early generations of the radish, the same result obtained by Koo [22], while only 2 pairs of 45S rDNA signals were detected in later generations (see Additional file 3). It suggests that rDNA also have a rapid evolution in the genome. Furthermore radish inbred lines from different areas might contain varied ratio of repetitive sequences. 30.73% of the 0713D genome is repetitive DNA in our study, while repetitive sequences occupied 26.6% of the Japanese radish ‘Aokubi’ genome [25].Compositions of each type of repetitive elements are also different between these two radishes. Overall, these results demonstrate the highly dynamic nature of radish genome.

### Rapid evolution of centromere sequence

The centromeres of higher eukaryotes are rich in repetitive DNA sequences which include large arrays of satellite repeats and/or retrotransposon-related repetitive sequences [8,9]. It has been shown that one single major satellite repeat is the dominating sequence in all centromeres in most diploid species [8,9]. In our study, the similar pattern of one type of centromeric satellite repeat (CL1) was detected by immunostaining and the ChIP test. However, it has been reported that some plant and animal species contain multiple satellite repeats associated with centromeres, such as in the common bean [44], potato [13], and chicken [45]. Centromeric satellite repeats diverge rapidly across species and often do not share any sequence similarity [8]. Several centromeric repeats were identified in potato and its closely related wide species *S. verrucosum*, respectively. Nevertheless, only one single homoeologous centromeric sequence was detected between these two species. This means centromeric regions of *Solanum* species show rapid evolution.

Taxonomic studies and rDNA gene space sequence analysis demonstrated that genus *Brassica* is a close relative of the genus *Raphanus* [46,47]. Our results also proved this. In this study, a new satellite CL25 was detected, which is distributed in radish and all tested *Brassica* species and located at the subtelomeric region of all tested species (Figure 2). Even in closely related species, centromeric satellites go through rapid evolution. CL1, the centromeric satellite repeat, shares high similarities with CentBr1 and CentBr2 sequences. These CentBr sequences appeared in the A and C genomes of *Brassica* species, while the corresponding centromeric repeats have not yet been identified in the B genome. Even in the same species, CentBr1 and CentBr2 have different distribution patterns on chromosomes [26]. These results indicate that centromeric satellite repeats of *Cruciferae* species evolve rapidly.

## Conclusions

In this study, we used low-coverage sequencing on *Raphanus sativus* L. (2n = 18) to analyze repeat elements. We revealed the genome structure of radish and found that satellite repeats are most dominating elements, which is differ from most reported species, in which LTR-retrotransposons are the most abundant element of the genome. The fine karyotyping analysis using satellites and rDNAs as markers allow us to easily identify each individual somatic metaphase chromosome. Only one putative *CENH3* (*RsCENH3*) gene was characterized and cloned from radish. Its deduced amino acid sequence shares high similarities to those of the CENH3s in *Brassica* species. In addition, Immunofluorescence- and ChIP-based assays demonstrated the functional significance of satellite and centromere-specific retrotransposon at centromeres. Our study provides a valuable basis for future genomic studies in radish.

### Availability of supporting data

The data sets supporting the results of this article are available in the NCBI SRA archive (accession no. SRX957720).

## Methods

### Plant materials

0713D (2n = 2x = 18, R genome), a Chinese *Raphanus sativus* L. inbred line, was used for Solexa genome sequencing, ChIP and cytogenetic studies. Plants were grown in the greenhouse with 16 hours in lights and 8 hours in the dark.

### Genomic DNA isolation and Solexa sequencing

DNA was isolated from 5 g of fresh young plant as described previously [48]. DNA was treated with DNase-free-RNase A for 3 h at RT for removing RNA, and purified by phenol/chloroform precipitation. Pellets were resuspended to a final concentration of 200–300 ng/μl. The sequencing was performed by HiSeq2000 platform (BerryGenomics. Beijing, China). One hundred bp paired-end reads were obtained from the results.

### Data analysis

Following a removal of linker/primer contaminations and artificially duplicated reads, a set of 5Gb whole genome Illumina paired end reads (Average length of reads was 100 bp), representing about 4.8× genome equivalent of radish [24] were used for similarity-based clustering analysis [38]. The clustering analysis was performed using a read similarity cutoff of 90% over at least 70% of the shorter sequence length. Reads within individual clusters were assembled into contigs. Sequence-similarity searches of assembled contigs were done for finding out which type and family of repeats they present. Clusters containing satellite repeats were identified based on graphs and the presence of tandem repeats within assembled contig sequences. Satellite sequences were identified using the Tandem Repeat Finder [49]. Clusters corresponding to putative mitochondrial and plastid contaminations were identified by searching GenBank and eliminated. The genome proportion of each cluster was calculated as the percentage of reads.

### FISH and immunostaining

In the FISH procedure, mitotic chromosomes were prepared as follows: seeds were geminated on moist miracloth at 28°C in the dark for 2 days, root tips from radish were collected and treated with pressurized nitrous oxide for 90 min, fixed in 3:1 (100% ethanol: glacial acetic acid) Carnoy’s solution for 2 days at room temperature (25°C) and then stored at −20°C until used. Probes were prepared by PCR amplification from radish genomic DNA with specific primers (see Additional file 5). The amplified DNAs were labeled with bio-16-UTP, digoxigenin-11-dUTP or DEAC (Roche. Basel, Switzerland) using a standard nick translation reaction. The FISH experiments, including slide pre-treatment, probe hybridization and signal detection were performed as reported according to published protocols [17]. Chromosomes were counterstained with 4′, 6-diamidino-2-phenylindole (DAPI) (Vector Laboratories. Burlingame, USA). Images were captured digitally using a Sensys CCD camera (QIMAGING, RETIGA-SRV, FAST 1394) attached to an Olympus BX61 epifluorescence microscope (Olympus. Tokyo, Japan). Images were adjusted with Adobe Photoshop 5.0. In order to draw an integrated ideogram of radish metaphase chromosomes, chromosomes in 5 metaphase cells were measured.

In the immunostaining procedure, root tips were fixed in freshly prepared 4% (w/v) paraformaldehyde solution for 30 min on ice and then washed three times for 10 min in 1× PBS (10 mM sodium phosphate, pH 7.0, and 140 mM NaCl) on ice. After washing with 1× PBS, the root tips were directly squashed on slides coated with poly-L-lysine. After removal of the cover slip, the slides were immersed in 1× PBS. The slides were incubated for 3 h at 37°C in a moist chamber with the mouse primary sera antibody against brassica CENH3 diluted in 1× TNB buffer. Following three rounds of washing in 1× PBS, anti-mouse-Alexa 488 diluted in 1:100 was applied for 1 h at 37°C. After three rounds of washing in 1× PBS, the slides were dried at room temperature. For detection of the CENH3 proteins, the chromosomes were counterstained with DAPI. For a combined detection of the CENH3 proteins and the satellite repeats, the slides were fixed in 4% (w/v) paraformaldehyde solution for 5 min and washed in 1× PBS for three times, then the FISH procedure was followed as usual.

### ChIP and quantitative ChIP-PCR

ChIP using the BrCENH3 antibody was performed on radish nucleosomes as previously described [50]. Approximately 10 g of 10-days-old radish plants were used for isolating nuclei. The isolated nuclei were suspended in 3 ml micrococcal nuclease (MNase) buffer (10% sucrose, 50 mM Tris–HCl Ph 7.5, 4 mM MgCl_2_, and 1 mM CaCl_2_) and then digested with micrococcal nuclease (Sigma) to produce a chromatin solution. The digested chromatin was used for ChIP experiments using the BrCENH3 antibody, and normal mouse serum was used as a mock treatment. Chromatin with the antibody was incubated with rotation overnight at 4°C. DNA from the ChIP and input control samples was diluted in 1× TE.

Quantitative real-time PCR analysis of pelleted DNA was used to determine the relative fold enrichment (RFE) of specific sequences within anti-BrCENH3 precipitated DNA relative to the DNA sample prepared from pre-blood immunoprecipitation. We used the CL25, which is located at the chromosome ends, as a negative control to normalize enrichment of each positive amplicon. Each sample had three replicates. 5S rDNA and 45S rDNA, which were not localized at centromere region, were also used for evaluating reliability of the results. Primers CL25-1L, CL25-1R, CL25-2L, CL25-2R, 5SL, 5SR, 45SL, 45SR, CL1-1L, CL1-1R, CL1-2L, CL1-2R, CR-Radish-1L, CR-Radish-1R, CR-Radish-2L and CR-Radish-2R were used for real-time PCR and are listed in Additional file 1: Table S2. The relative expression levels were calculated according to cycle number. Quantitative PCR data were performed as described previously [28].

### Cloning of CENH3 cDNA

To identify radish CENH3 orthologs sequences, the *BrCENH3* complementary cDNA sequence (GenBank accession number GU166737.1), as the query, was searched by NCBI BLAST. Two radish CENP-A genes sequence were identified. Total RNA was extracted from leaf tissue of an inbred line ‘0713D’. RNA samples were treated with RNase-free DNase (Promega. Madison, USA) and dissolved in RNase-free double-distilled water. cDNA was synthesized using the RNA and Superscript III RT (Invitrogen, Carlsbad, USA). The primers CENH3-L and CENH3-R were used for amplification of full length CDS of *CENH3*. The fragments were cloned and sequenced. Multiple sequence alignment of CENH3 was performed using MUSCLE [51].

## Additional files

Additional file 1:
**List of the annotation and genome proportion of clusters.**
Additional file 2:
**FISH mapping of CL43 repeats in radish.**
Additional file 3:
**FISH mapping of 45S rDNA in different generation of radish.** (a) Early generation; (b) Later generation. Additional file 4:
**ChIP-FISH using BrCENH3 antibodies.** (a) FISH signals derived from ChIP using normal mouse serum; (b) FISH signals derived from ChIP using anti-CENH3 antibodies. Additional file 5:
**Primers used in this study.**

## Abbreviations

FISH: Fluorescent in situ hybridization
Gb: Giga base pairs
Mb: Mega base pairs
bp: Base pair
ChIP: Chromatin immunoprecipitation
