# Supplementary material for: Repetitive sequence analysis and karyotyping reveals centromere-associated DNA sequences in radish (Raphanus sativus L.)
Source: BMC Plant Biol. 2015 Apr 18;15:105. doi: 10.1186/s12870-015-0480-y (PMC4417506; doi:10.1186/s12870-015-0480-y)
Supplement: Additional file 1: — List of the annotation and genome proportion of clusters. [file 12870_2015_480_MOESM1_ESM.docx]

**List of the annotation and genome proportion of clusters**

|  | **cluster** | **Genome proportion [%]** | | **Annotation** |
| --- | --- | --- | --- | --- |
| **1** | **CL1** | **8.68** | **Satellite** | |
| **2** | **CL2** | **3.64** | **Satellite** | |
| **3** | **CL3** | **1.35** | **LTR.Copia** | |
| **4** | **CL4** | **1.14** | **LTR.Copia** | |
| **5** | **CL6** | **0.893** | **DNA.hAT.Ac** | |
| **6** | **CL7** | **0.883** | **rRNA** | |
| **7** | **CL8** | **0.873** | **LTR.Gypsy** | |
| **8** | **CL9** | **0.862** | **LTR.Copia** | |
| **9** | **CL10** | **0.846** | **LTR.Gypsy** | |
| **10** | **CL12** | **0.828** | **rRNA** | |
| **11** | **CL14** | **0.783** | **LTR.Copia** | |
| **12** | **CL15** | **0.699** | **LTR.Copia** | |
| **13** | **CL17** | **0.669** | **rRNA** | |
| **14** | **CL19** | **0.649** | **rRNA** | |
| **15** | **CL20** | **0.634** | **rRNA** | |
| **16** | **CL21** | **0.615** | **LTR.Gypsy** | |
| **17** | **CL22** | **0.547** | **LTR.Copia** | |
| **18** | **CL24** | **0.445** | **LTR.Gypsy** | |
| **19** | **CL25** | **0.439** | **Satellite** | |
| **20** | **CL29** | **0.417** | **rRNA** | |
| **21** | **CL36** | **0.237** | **LTR.Gypsy** | |
| **22** | **CL37** | **0.205** | **LINE.L1** | |
| **23** | **CL38** | **0.205** | **LTR.Gypsy** | |
| **24** | **CL39** | **0.199** | **LTR.Copia** | |
| **25** | **CL40** | **0.196** | **LTR.Gypsy** | |
| **26** | **CL42** | **0.183** | **rRNA** | |
| **27** | **CL43** | **0.173** | **Satellite** | |
| **28** | **CL45** | **0.157** | **DNA.MULE.MuDR** | |
| **29** | **CL46** | **0.15** | **unclasified** | |
| **30** | **CL48** | **0.13** | **LTR.Gypsy** | |
| **31** | **CL49** | **0.112** | **DNA.MULE.MuDR** | |
| **32** | **CL50** | **0.109** | **LTR.Gypsy** | |
| **33** | **CL52** | **0.105** | **LTR.Gypsy** | |
| **34** | **CL53** | **0.09** | **LINE.L1** | |
| **35** | **CL54** | **0.079** | **LTR.Gypsy** | |
| **36** | **CL57** | **0.061** | **unclasified** | |
| **37** | **CL58** | **0.059** | **DNA.TcMar.Stowaway** | |
| **38** | **CL59** | **0.057** | **LTR.Gypsy** | |
| **39** | **CL60** | **0.053** | **LTR.Gypsy** | |
| **40** | **CL61** | **0.05** | **unclasified** | |
| **41** | **CL62** | **0.049** | **LTR.Gypsy** | |
| **42** | **CL63** | **0.048** | **LTR.Gypsy** | |
| **43** | **CL64** | **0.048** | **DNA.CMC.EnSpm** | |
| **44** | **CL65** | **0.047** | **LTR.Gypsy** | |
| **45** | **CL66** | **0.046** | **unclasified** | |
| **46** | **CL67** | **0.046** | **LTR.Gypsy** | |
| **47** | **CL68** | **0.045** | **LTR.Copia** | |
| **48** | **CL69** | **0.045** | **LTR.Gypsy** | |
| **49** | **CL70** | **0.041** | **LTR.Copia** | |
| **50** | **CL71** | **0.04** | **LTR.Gypsy** | |
| **51** | **CL72** | **0.04** | **rRNA** | |
| **52** | **CL73** | **0.037** | **LTR.Gypsy** | |
| **53** | **CL74** | **0.035** | **LTR.Gypsy** | |
| **54** | **CL75** | **0.035** | **unclasified** | |
| **55** | **CL76** | **0.034** | **unclasified** | |
| **56** | **CL77** | **0.034** | **unclasified** | |
| **57** | **CL78** | **0.033** | **LTR.Copia** | |
| **58** | **CL79** | **0.033** | **LTR.Gypsy** | |
| **59** | **CL80** | **0.033** | **unclasified** | |
| **60** | **CL81** | **0.033** | **unclasified LTR** | |
| **61** | **CL82** | **0.032** | **LTR.Gypsy** | |
| **62** | **CL83** | **0.032** | **LTR.Gypsy** | |
| **63** | **CL84** | **0.03** | **unclasified** | |
| **64** | **CL85** | **0.03** | **LTR.Gypsy** | |
| **65** | **CL86** | **0.029** | **LTR.Gypsy** | |
| **66** | **CL87** | **0.029** | **LTR.Gypsy** | |
| **67** | **CL88** | **0.029** | **LTR.Gypsy** | |
| **68** | **CL90** | **0.028** | **unclasified** | |
| **69** | **CL91** | **0.027** | **unclasified** | |
| **70** | **CL92** | **0.026** | **LTR.Gypsy** | |
| **71** | **CL93** | **0.026** | **LTR.Gypsy** | |
| **72** | **CL94** | **0.026** | **LTR.Copia** | |
| **73** | **CL95** | **0.026** | **LTR.Gypsy** | |
| **74** | **CL96** | **0.023** | **unclasified** | |
| **75** | **CL97** | **0.023** | **unclasified** | |
| **76** | **CL98** | **0.023** | **unclasified** | |
| **77** | **CL99** | **0.023** | **DNA.CMC.EnSpm** | |
| **78** | **CL100** | **0.022** | **DNA.TcMar.Stowaway** | |
| **79** | **CL101** | **0.022** | **unclasified** | |
| **80** | **CL102** | **0.021** | **LTR.Gypsy** | |
| **81** | **CL103** | **0.021** | **unclasified** | |
| **82** | **CL104** | **0.021** | **LTR.Gypsy** | |
| **83** | **CL105** | **0.021** | **LINE.L1** | |
| **84** | **CL106** | **0.02** | **LTR.Copia** | |
| **85** | **CL107** | **0.02** | **unclasified** | |
| **86** | **CL108** | **0.02** | **LTR.Gypsy** | |
| **87** | **CL109** | **0.019** | **LTR.Gypsy** | |
| **88** | **CL110** | **0.019** | **unclasified** | |
| **89** | **CL112** | **0.018** | **unclasified** | |
| **90** | **CL114** | **0.018** | **unclasified** | |
| **91** | **CL115** | **0.017** | **LTR.Gypsy** | |
| **92** | **CL116** | **0.017** | **unclasified** | |
| **93** | **CL117** | **0.016** | **unclasified** | |
| **94** | **CL118** | **0.016** | **LTR.Copia** | |
| **95** | **CL120** | **0.016** | **LTR.Copia** | |
| **96** | **CL121** | **0.016** | **rRNA** | |
| **97** | **CL122** | **0.016** | **unclasified** | |
| **98** | **CL123** | **0.015** | **LTR.Gypsy** | |
| **99** | **CL124** | **0.015** | **DNA.CMC.EnSpm** | |
| **100** | **CL125** | **0.015** | **unclasified** | |
| **101** | **CL126** | **0.015** | **LTR.Gypsy** | |
| **102** | **CL127** | **0.015** | **DNA.hAT.Ac** | |
| **103** | **CL128** | **0.015** | **LTR.Gypsy** | |
| **104** | **CL129** | **0.015** | **unclasified** | |
| **105** | **CL130** | **0.015** | **LTR.Gypsy** | |
| **106** | **CL132** | **0.015** | **unclasified** | |
| **107** | **CL133** | **0.015** | **unclasified** | |
| **108** | **CL134** | **0.015** | **LTR.Copia** | |
| **109** | **CL135** | **0.014** | **Unclasified** | |
| **110** | **CL136** | **0.014** | **unclasified** | |
| **111** | **CL137** | **0.014** | **unclasified** | |
| **112** | **CL138** | **0.014** | **DNA.hAT.Ac** | |
| **113** | **CL140** | **0.014** | **DNA.TcMar.Pogo** | |
| **114** | **CL141** | **0.014** | **LTR.Gypsy** | |
| **115** | **CL142** | **0.014** | **LTR.Gypsy** | |
| **116** | **CL143** | **0.013** | **LTR.Copia** | |
| **117** | **CL144** | **0.013** | **LTR.Copia** | |
| **118** | **CL145** | **0.013** | **LTR.Gypsy** | |
| **119** | **CL146** | **0.013** | **LTR.Gypsy** | |
| **120** | **CL147** | **0.012** | **DNA.CMC.EnSpm** | |
| **121** | **CL149** | **0.012** | **SINE.tRNA** | |
| **122** | **CL150** | **0.012** | **unclasified** | |
| **123** | **CL151** | **0.012** | **LTR.Gypsy** | |
| **124** | **CL152** | **0.012** | **unclasified** | |
| **125** | **CL153** | **0.012** | **unclasified** | |
| **126** | **CL154** | **0.011** | **unclasified** | |
| **127** | **CL155** | **0.011** | **unclasified** | |
| **128** | **CL156** | **0.011** | **unclasified** | |
| **129** | **CL157** | **0.011** | **LTR.Gypsy** | |
| **130** | **CL158** | **0.011** | **unclasified** | |
| **131** | **CL159** | **0.011** | **LTR.Gypsy** | |
| **132** | **CL160** | **0.011** | **LTR.Gypsy** | |
| **133** | **CL161** | **0.011** | **LTR.Gypsy** | |
| **134** | **CL162** | **0.011** | **DNA.TcMar.Stowaway** | |
| **135** | **CL163** | **0.011** | **unclasified** | |
| **136** | **CL164** | **0.011** | **unclasified** | |
| **137** | **CL165** | **0.011** | **DNA.hAT.Ac** | |
| **138** | **CL166** | **0.011** | **unclasified** | |
| **139** | **CL167** | **0.011** | **LTR.Gypsy** | |
| **140** | **CL168** | **0.01** | **unclasified** | |
| **141** | **CL169** | **0.01** | **unclasified** | |
| **142** | **CL171** | **0.01** | **LTR.Gypsy** | |
| **143** | **CL172** | **0.01** | **unclasified** | |
| **144** | **CL173** | **0.01** | **unclasified** | |
