# Supplementary figures and images for: Repetitive sequence analysis and karyotyping reveals centromere-associated DNA sequences in radish (Raphanus sativus L.)
Source: BMC Plant Biol. 2015 Apr 18;15:105. doi: 10.1186/s12870-015-0480-y (PMC4417506; doi:10.1186/s12870-015-0480-y)

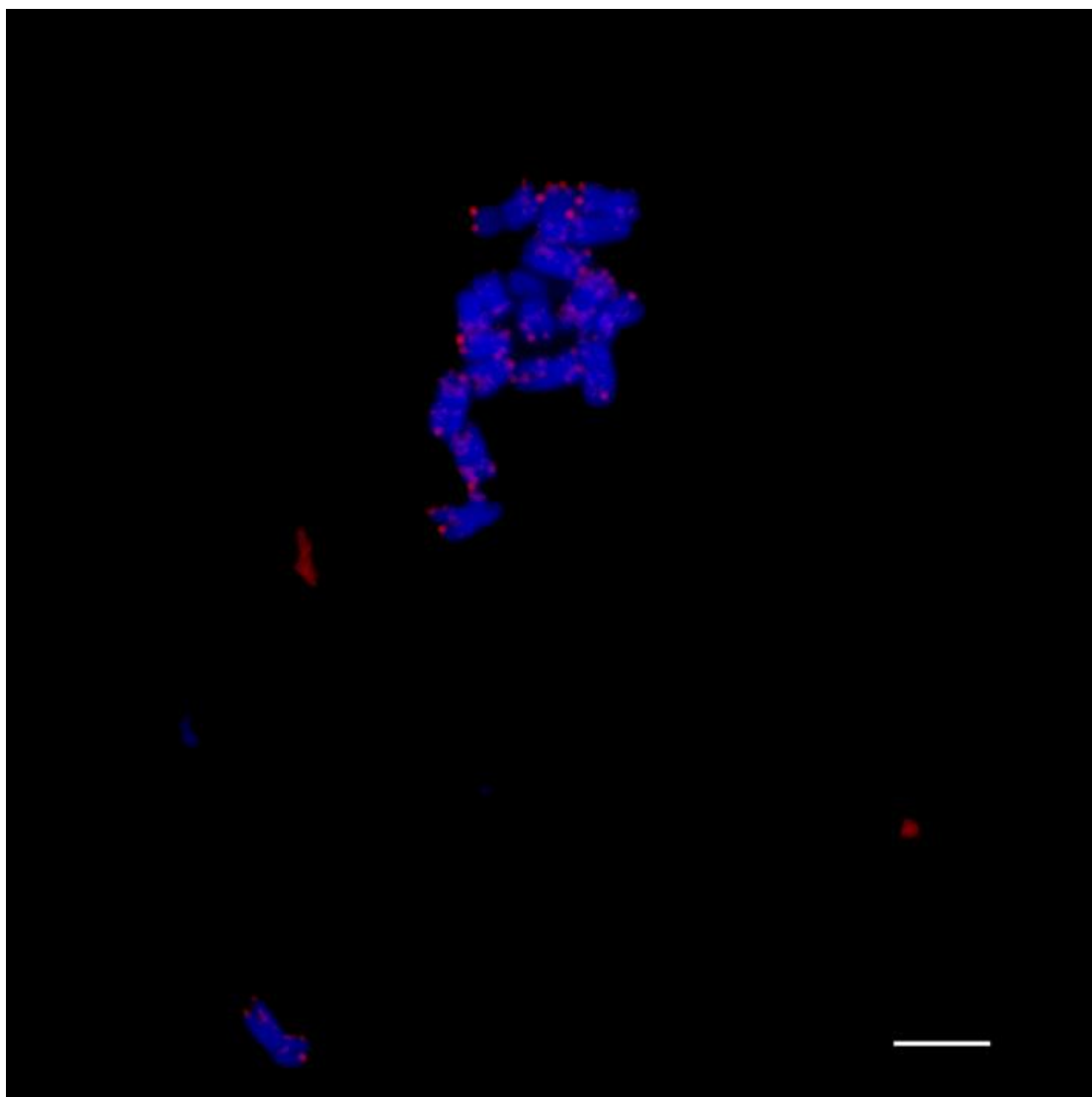

Supplement: Additional file 2: — FISH mapping of CL43 repeats in radish. [file 12870_2015_480_MOESM2_ESM.pdf]

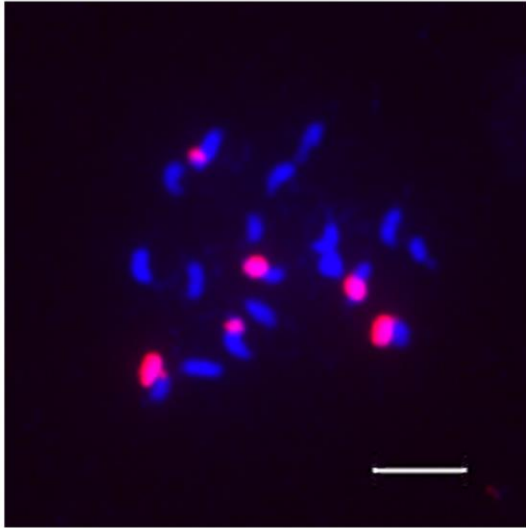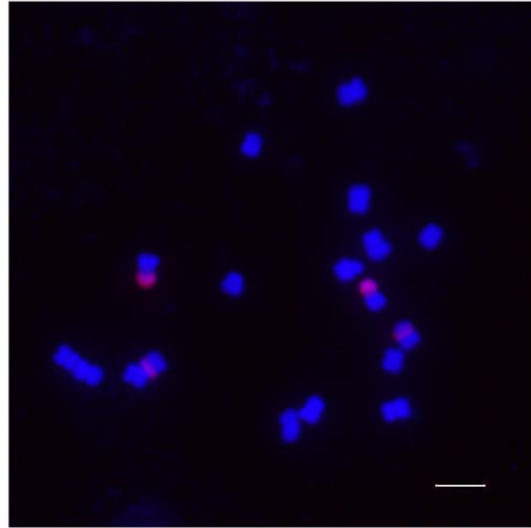

Supplement: Additional file 3: — FISH mapping of 45S rDNA in different generation of radish. (a) Early generation; (b) Later generation. [file 12870_2015_480_MOESM3_ESM.pdf]

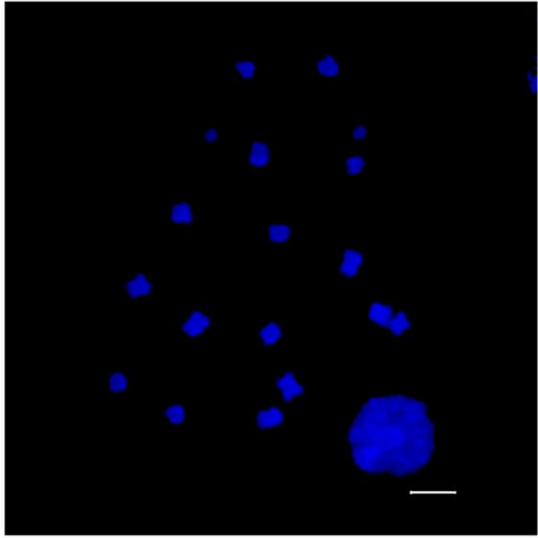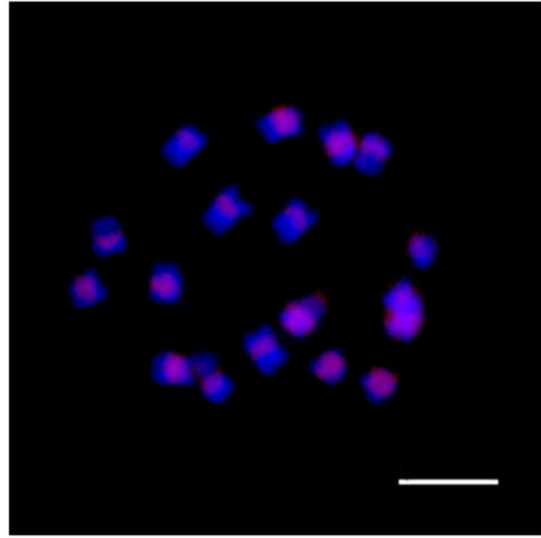

Supplement: Additional file 4: — ChIP-FISH using BrCENH3 antibodies. (a) FISH signals derived from ChIP using normal mouse serum; (b) FISH signals derived from ChIP using anti-CENH3 antibodies. [file 12870_2015_480_MOESM4_ESM.pdf]
