# Supplementary material for: Repetitive sequence analysis and karyotyping reveals centromere-associated DNA sequences in radish (Raphanus sativus L.)
Source: BMC Plant Biol. 2015 Apr 18;15:105. doi: 10.1186/s12870-015-0480-y (PMC4417506; doi:10.1186/s12870-015-0480-y)
Supplement: Additional file 5: — Primers used in this study. [file 12870_2015_480_MOESM5_ESM.docx]

**Primers used in this study**

| **Primer name** | **Primer Sequence** | **Mark** |
| --- | --- | --- |
| **CL1-L** | **TGGTTTCAAATCAAGCTTCTTACAT** | **FISH probe** |
| **CL1-R** | **TCAAACCAGAAGGAACAAAGATG** |  |
| **CL25-L** | **TGTCAGTGCTCGATGAAACT** | **FISH probe** |
| **CL25-R** | **CGAAAACTCAATATTTTTGAAGGGG** |  |
| **CL25-1L** | **GGCCAATTCAACTAATCTATGCAGT** | **Realtime PCR** |
| **CL25-1R** | **TTCAAACCACATTCGACATCTGAAA** |  |
| **CL25-2L** | **AGTGTATAGTTTCATCGAGCACT** |  |
| **CL25-2R** | **AGAGAAAAGGAGACAACAAAGGT** |  |
| **CR-Radish-1L** | **TCGACCAGAGGGAGTGAGAG** |  |
| **CR-Radish-1R** | **TGGACGTGGGAAACACTGAG** |  |
| **CR-Radish-2L** | **CTTGGGCAAGCTTGGAACTG** |  |
| **CR-Radish-2R** | **TAAACCCACTGGCCAATCCC** |  |
| **CL1-1L** | **TGGTTTCAAATCAAGCTTCTTACAT** |  |
| **CL1-1R** | **TCAAACCAGAAGGAACAAAGATG** |  |
| **CL1-2L** | **AGGAACCACGATGTAAGAAGC** |  |
| **CL1-2R** | **GGATAGTTCTTCCTCATAACGCCT** |  |
| **45S-L** | **GATGGGACAGTCAGGGTGTG** |  |
| **45S-R** | **GGACAGTCTGCGGGGTATTT** |  |
| **5S-L** | **TTTTCGTCGGAGAGCACGAG** |  |
| **5S-R** | **TACGGGCTGTGAACGAGATG** |  |
| **CENH3-L** | **ATGGCGAGAACGAAGCATTTC** | **Amplification of CENH3** |
| **CENH3-R** | **TCAAAAGGGTCTGCCTTTTCCT** |  |
